# Expression of Recombinant Human Lysozyme in Egg Whites of Transgenic Hens

**DOI:** 10.1371/journal.pone.0118626

**Published:** 2015-02-23

**Authors:** Dainan Cao, Hanyu Wu, Qingyuan Li, Yingmin Sun, Tongxin Liu, Jing Fei, Yaofeng Zhao, Sen Wu, Xiaoxiang Hu, Ning Li

**Affiliations:** State Key Laboratory for Agrobiotechnology, College of Biological Sciences, China Agricultural University, Beijing, 100193, P. R. China; Duke University Medical Center, UNITED STATES

## Abstract

Chicken egg lysozyme (cLY) is an enzyme with 129 amino acid (AA) residue enzyme. This enzyme is present not only in chicken egg white but also in mucosal secretions such as saliva and tears. The antibacterial properties of egg white can be attributed to the presence of lysozyme, which is used as an anti-cancer drug and for the treatment of human immunodeficiency virus (HIV) infection. In this study, we constructed a lentiviral vector containing a synthetic cLY signal peptide and a 447 bp synthetic human lysozyme (hLY) cDNA sequence driven by an oviduct-specific ovalbumin promoter, and microinjected into the subgerminal cavity of stage X chick embryos to generate transgenic chicken. The transgene inserted in the chicken chromosomes directs the synthesis and secretion of hLY which has three times higher specific activity than cLY. Three G_1_ transgenic chickens were identified, the only female of which expressed recombinant human lysozyme (rhLY) at 57.66 ± 4.10 μg/ml in the egg white and the G_2_ transgenic hens of the G_1_ transgenic cock A011 expressed rhLY at 48.72 ± 1.54 μg/ml. This experiment demonstrated that transgenic hens with stable oviduct-specific expression of recombinant human lysozyme proteins can be created by microinjection of lentiviral vectors. The results of this research could be contribute to the technological development using transgenic hens as a cost-effective alternative to other mammalian systems, such as cow, sheep and goats, for the production of therapeutic proteins and other applications.

## Introduction

Lysozymes (LYs), which are muramidases/N-acetylmuramide glycanhydrolases, are found widely in milk, saliva, and tears in mammals and also in avian egg white. In the peptidoglycan of bacterial cell walls and the chitin of fungal cell walls, LY catalyses the hydrolysis of 1,4-β-*N*-acetylglucosamine linkages between the C-1 of *N*-acetylmuramic acid and the C-4 of *N*-acetylglucosamine[1, 2]. Due to its wide-ranging ability to lyse the cell walls of various microorganisms, LY functions as a crucial bio-defence factor in innate immunity. Numerous reports have demonstrated that LY plays important roles in protecting against bacteria, viruses and fungi [3–6], and the use of LY as a preservative in the food industry has been well established.

**Fig 1:**
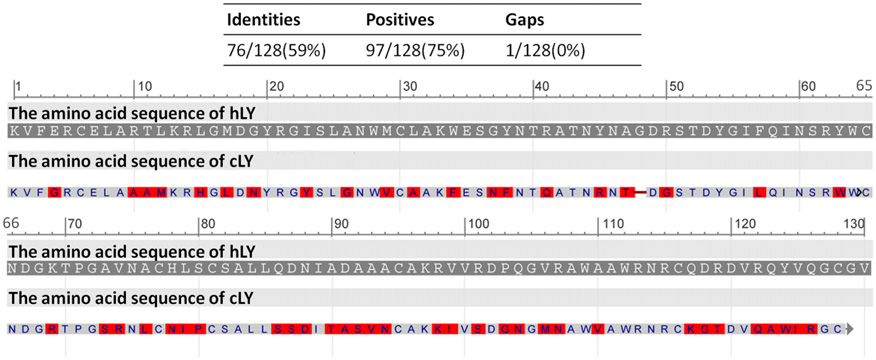
The alignment of the amino acid sequences of cLY and hLY protein. Red highlighted residues above the sequences represent the amino acid differences between the cLY and hLY protein. The alignment of the amino acid sequences was analysed using the Protein Blast (http://blast.ncbi.nlm.nih.gov/).

Based on differences in structural, catalytic and immunological characteristics, LYs have been classified into three major types: chicken-type (c-type), goose-type (g-type) and invertebrate-type (i-type) LYs. In addition, several other types of LYs, including phage-type, bacterial-type and plant-type LYs, among others, have been identified [1, 7, 8]. Both cLY and hLY are c-type LYs. cLY is composed of 129 amino acid residues and has a molecular weight of 14.3 kDa, whereas hLY is composed of 130 amino acid residues and has a molecular weight of 14.7 kDa. The cLY protein shares 59% identity in acids with hLY protein (Fig. 1). Despite the similarities between these two LYs with respect to number of amino acids and molecular weight, the anti-bacterial activity of hLY is approximately threefold greater than the anti-bacterial activity of cLY.

Different recombinant human lysozyme concentrations have reportedly been expressed in transgenic microorganisms [9,10], plants [11, 12] and animals [13, 14]. In addition, studies have demonstrated that rhLY can enhance the immunity of transgenic organisms and improve the prevention of invasive pathogens. Transgenic tobacco plants exhibit enhanced resistance to the fungus *Erysiphe cichoracearum* [11]. Moreover, the growth of not only mastitis-causing strains of *Escherichia coli* and *Staphylococcus aureus* but also the cold-spoilage organism *Pseudomonas fragi* was slower in milk from transgenic goats than in milk from unmodified goats [15]. In our prior work, we have successfully produced transgenic mice [16, 17], pigs [18] and cattle [19] with milk-specific rhLY expression. In this study, we report the production of transgenic chickens with the specific expression of rhLY in the oviducts of laying hens.

A new laid fertilized egg is a chick embryo which has already contained about 60,000 cells because of the unique feature of chicken reproductive system. The avian egg, with its solid sealed eggshell and large fragile yolk, is not as transparent as mammalian ova, making it a challenge to view for injection and manipulation. In 1988, Perry developed a complete in vitro culture system supporting the chick embryos from the fertilized ovum to hatching [20], allowing for a better accessibility for the manipulation of the embryos. A few years later, Helen Sang’s group first produced germ-line transgenic chickens through the microinjection of linearized gene constructs into the newly fertilized zygote based on Perry’s surrogate eggshell culture system, although the germ-line transmission frequency was low [21]. Using replication-deficient retroviral vectors, such as avian leukosis virus-based vectors [22] and avian spleen necrosis virus-based vectors [23], have been shown capable of producing chimeric transgenic birds, but the transmission frequencies were still low (about 0.7% to 0.9%). In the recent decades, with the widely application of the lentivirus and the successful culture of chicken primordial germ cells (PGCs), efforts for manipulating the avian genome have focused on using viral vectors [24–27] and using PGCs [28–30], resulting in founder birds transmitting the exogenous gene to as much as 52.2%.

Several recombinant proteins have been reported successfully expressed in the white of a laid egg, including bacterial β-lactamase [22], human interferon α-2b [31], human monoclonal antibody [32], single-chain Fv-Fc fusion protein [33] and two therapeutic proteins [34]. Here we report successful expression of recombinant human lysozyme in the egg white of transgenic hens using the lentivirus as an exogenous gene deliverer.

## Materials and Methods

### Plasmid construction

The plasmid pWPXL was purchased from Addgene (Cambridge, USA). This plasmid contains a 5’ long terminal repeat (LTR), a 3’ self-inactivating LTR, the HIV-1 packaging signal, an EF1-alpha promoter, an enhanced green fluorescent protein (eGFP) reporter gene and a woodchuck hepatitis virus post-transcriptional regulatory element (WPRE). A 2.8-kb chicken ovalbumin promoter (OV, Data A in S1 Data) was cloned by PCR, using the primers 2.8kOV-F and 2.8kOV-R (S1 Table). The 2.8-kb ovalbumin promoter contained the exon 1, intron 1, and the beginning of exon 2 of the chicken ovalbumin gene (Gene ID: 396058) which produces about half of the protein of chicken egg whites. A 673-bp oestrogen-responsive enhancer element (ERE, Data B in S1 Data) which locates in the 5’ flank region of the chicken ovalbumin gene, was cloned by PCR, using the primers ERE-F and ERE-R. The cDNA sequence of human lysozyme was modified in accordance with chicken codon bias, and a signal peptide from the chicken egg white lysozyme was added to the 5’ end of this sequence; the resulting sequences were synthesized by Sangon Biotech (Shanghai, China).

### Cell lines

Cell lines used in this study, including human fibrosarcoma cells HT1080, human embryonic kidney cells HEK293FT, human embryonic kidney cells HEK293T, baby hamster kindey cells BHK and chicken embryo fibroblast cells DF-1, were from the Cell Preservation Centre of our laboratory.

### Cell culture and lentivirus production

HEK293FT cells were grown at 37°C and 5% CO_2_ in Dulbecco’s modified Eagle’s medium (DMEM) supplemented with 10% (v/v) foetal bovine serum (FBS), 2 mM glutamine, 100 μg/ml streptomycin and 100 U/ml penicillin (Invitrogen, Carlsbad, USA). For transduction, 3.5×10^6^ cells were seeded into a 100-mm dish, and 600 μl Opti-MEM premixed with 20 μl of FuGENE HD Transfection Reagent (Roche, South San Francisco, USA), 1.2 μg of vector plasmid (pWPXL-EREOV-hLY), 2.4 μg of gag/pol plasmid (psPAX2, Addgene) and 0.5 μg of VSV-G plasmid (pMD2.G, Addgene) was dispersed dropwise into the culture medium when cells reached 60–70% confluence. At 24–48 h after transfection, culture supernatants were filtered (using a 0.22-μm filter) and then centrifuged at 25,000×*g* for 12 h at 4°C. The virus pellet was resuspended in PBS and subsequently subjected to ultracentrifugation at 50,000×*g* for 2 h at 4°C. The virus was resuspended in virus preservation solution, aliquoted and stored at -80°C.

### Virus titre determination

Human fibrosarcoma HT1080 cells were transduced with a packaged lentiviral construct in a 24-well plate (Corning, New York, USA). Three days after infection, the culture medium was removed, and the cells in each well were washed with 1 ml PBS, lysed and transferred into a PCR tube. The resulting tubes were then heated at 95°C for 2 min, centrifuged at 16,000×*g* for 1 min and stored at 4°C. Virus titres were determined using the UltraRapid Lentiviral Titer Kit (System Biosciences, Mountain View, USA).

### Microinjection of the lentiviral vector and egg incubation

Freshly laid White Leghorn eggs were purchased from the China Agricultural University Ranchette (Beijing, China). The upper surfaces of the eggs were washed in a 0.1% benzalkonium bromide solution at 40°C for 3 min. A microscopic manipulator (Eppendorf, Hamburg, Germany) was used to inject approximately 1–2 μl of lentiviral vector into each sub-germinal cavity, beneath the blastodermal embryo. Embryos were placed into surrogate eggshells and incubated at 37.5°C and 60–70% relative humidity (RH) for approximately 3 days (until stage HH16–17), with a 90° rotation every 30 minutes; subsequently, these embryos were transferred into new, larger surrogate eggshells and incubated at 37.5°C and 60–70% RH for 17 additional days, with a 30° rotation every 30 minutes [20]. At E20, the embryos were transferred to a hatching incubator for continued incubation at 37°C and 50% RH.

### Polymerase chain reaction analysis

Genomic DNA from embryos that died during the culture process, heart and ovarian tissues from two-month-old G_0_ hens, semen from mature G_0_ cockerels and blood from hLY-positive G_1_ birds were extracted by proteinase K digestion, phenol and chloroform extraction and ethanol precipitation. DNA from the cockscombs of G_1_ chicks was extracted according to the standard HotSHOT protocol [35], and 3 μl of the soultion were used to perform PCR analysis. In accordance with the manufacturer’s instructions, PCR analysis was performed with GoldStar Taq DNA Polymerase (CWBIO, Beijing, China), using a total reaction volume of 25 μl. The PCR amplification conditions were as follows: 94°C for 10 min, followed by 35 cycles of amplification (94°C for 30s, 60°C for 30 s, 72°C for 30 s) and one cycle of 72°C for 7 min. To identify the transgenic gene in G_0_ chimeric chickens, 500 ng of genomic DNA were added in each PCR reaction. In the PCR analyses of semen DNA from G_0_ males, control PCR reactions were performed in parallel, using 500 ng aliquots of chicken genomic DNA containing appropriate quantities of plasmid DNA to estimate approximate copy numbers. And 20 ng of genomic DNA were used for the amplification of the chicken housekeeping gene GAPDH. The primer pairs used (S1 Table) to identify the transgenic gene were hLY-F and hLY-R, the primers used to amplify the sequence of the chicken housekeeping gene GAPDH were cGAPDH-F and cGAPDH-R, and the primers used to identify the sex of the examined birds were Qsex-F and Qsex-R [30].

### RT-PCR amplification

Total RNA was extracted from chicken tissues using Trizol reagent (TianGen, Beijing, China), then 2 μg of RNA were used for the synthesis of cDNA with M-MLV reverse transcriptase (Promega, Madison, USA). Primers hLY-F and hLY-R were used for the amplification of the hLY genes and the primer pairs RT-cGAPDH-F and RT-cGAPDH-R which were designed to span an intron were used for the amplification of the chicken housekeeping gene GAPDH (S1 Table). RT-PCR amplification conditions were as follows: 94°C for 5 min, followed by 25 cycles of amplification (94°C for 30s, 55°C for 30 s, 72°C for 30 s) and one cycle of 72°C for 7 min.

### Southern blot analysis

Genomic DNA (10 μg) from the blood of G_1_ transgenic and non-transgenic birds was digested using *Pvu*II or *Nde*I to generate either a fragment spanning the vector or a junction fragment between viral and chicken genomic sequences respectively, then separated on a 0.8% agarose gel and transferred to a Hybond-N membrane (Amersham Pharmacia Biotech, Piscataway, USA). Southern blot analysis was performed with specific hybridisation probes that had been labelled with digoxigenin (DIG) using the PCR DIG Probe Synthesis Kit (Roche). The hLY probe (produced using the primers HLY-F119 and HLY-R444; S1 Table) was used to assess experimental samples.

### Genome walking

To identify the transgene insertion site, three rounds of thermal asymmetric PCR were performed using a genome walking kit (Takara, Dalian, China). These PCR amplifications used a DNA sample (500 ng) from each G_1_ transgenic chicken; the three gene-specific primers WPRE-SP1, WPRE-SP2 and WPRE-SP1 (S1 Table); and the four random primers AP1, AP2, AP3 and AP4, which were supplied in the genome walking kit. The products of the third round of the genome walking PCR were purified using the E.Z.N.A. Gel Extraction Kit (Omega Bio-Tek, Norcross, USA) and then directly sequenced. The sequencing results were analysed using the BLAST database of assembled genomes (http://blast.ncbi.nlm.nih.gov) and the University of California Santa Cruz Genome Browser (http://www.genome.ucsc.edu).

### Immunofluorescence

The adult hen oviduct tissues were fixed in 4% paraformaldehyde in PBS for 3 h, followed by 15 min wash with PBS and incubated in 30% sucrose overnight at 4°C. Tissues were embedded in paraffin and sectioned to obtain 10 μm thickness. The sections were rehydrated and blocked for 2 h in blocking solution (ZSGB-BIO, Beijing, China). Mouse anti-Human Lysozyme antibody (US Biological, Swampscott, USA) was diluted 1:100 in antibody diluent (ZSGB-BIO) and incubated overnight at 4°C. Second antibody (Invitrogen, Carlsbad, USA) was at a 1:400 dilution and incubated at room temperature for 2 h. Samples were analyzed using Olympus Fluoview FV1000 confocal microscope (Olympus, Melville, USA) with the same parameters. Sections of the oviduct tissues of a wild type White Leghorn were dealt with by the same process as controls.

### Protein analysis

The whites of eggs from G_1_/G_2_ transgenic hens and wild-type White Leghorns (as controls) were mixed with 4 volumes of ice-cold 50 mM Na acetate buffer (pH 5.0) for 2 h at 4°C to remove the bulk of the ovomucin fraction. The resulting mixture was divided into aliquots and stored at -20°C for further analysis. Concentrations of human lysozyme protein in egg whites were determined using the ELISA Kit for Human Lysozyme (USCN, Wuhan, China).

### Animal euthanasia

All birds received standard diet and water. The experimental adult chickens were sacrificed after injected with sodium pentobarbital intravenously at a dose rate of at least 80mg/kg. The experimental non-transgenic G_1_ and G_2_ chicks were remained in chambers filled with more than 70% carbon dioxide for at least 15 min for euthanasia.

### Ethics statement

All animal work was conducted according to the guidelines for the care and use of experimental animals established by the Ministry of Science and Technology of the People’s Republic of China. The protocol was approved by the Institutional Animal Care and Use Committee of the China Agricultural University (Permit Number: SKLAB-2012–06–01). All surgery was performed under sodium pentobarbital anesthesia, and all efforts were made to minimize suffering.

## Results

### Plasmid preparation

**Fig 2:**
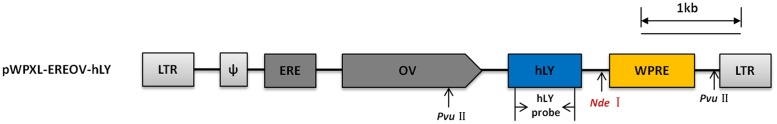
A schematic representation of the structure of the lentiviral vector used to generate G_0_ chimeric chickens. LTR, long terminal repeat; ψ, packaging signal; ERE, oestrogen responsive element; OV, chicken ovalbumin promoter; hLY, chicken egg white lysozyme signal peptide and the cDNA sequence of human lysozyme; WPRE, woodchuck hepatitis virus post-transcriptional regulatory element; The positions of *Pvu*II and *Nde*I restriction sites and the hybridisation probe used in Southern blot analysis are labelled.

The lentiviral vector pWPXL-EREOV-hLYwas used to generate G_0_ chimeric chickens. This plasmid contains the cDNA sequence of hLY, which has been modified at its 5’ end by the addition of a signal peptide from chicken egg white lysozyme; this modified hLY was driven by the 2.8-kb OV promoter. In the pWPXL-EREOV-hLY construct, a 673-bp oestrogen-responsive element (ERE) was cloned directly to the 5’ end of the OV. The cDNA sequence of human lysozyme was modified in accordance with chicken codon bias; moreover, a signal peptide from chicken egg white lysozyme was added to the 5’ end of this sequence (Fig. 2). The resulting modified hLY sequence was cloned into the plasmid pBudCE4.1 (Invitrogen) contains the human cytomegalovirus (CMV) immediate-early promoter and the human elongation factor 1α-subunit (EF-1α) promoter for independent expression of two proteins. This plasmid was transduced into four different cells: HEK293T, BHK, HT1080 and DF-1, allowing the modified hLY to be estimated by RT-PCR analysis and western blot analysis (S1 Fig.).

### The production and analysis of G_0_ chimeric chickens

**Fig 3:**
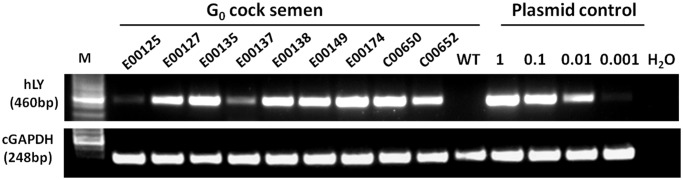
PCR analyses of semen DNA from G_0_ males with an integrated hLY fragment. To estimate approximate copy numbers, control PCR reactions were performed in parallel, using 500 ng aliquots of chicken genomic DNA containing appropriate quantities of plasmid DNA to simulate a single-copy gene (1); a 10-fold dilution of a single-copy gene (0.1); a 100-fold dilution of a single-copy gene (0.01); and a 1000-fold dilution of a single-copy gene (0.001). 20 ng of genomic DNA were used for the amplification of the chicken housekeeping gene GAPDH. WT, wild-type White Leghorns.

The aforementioned vectors were packaged and concentrated to produce virus titres of approximately 10^9^–10^11^ transducing units per millilitre (TU/ml). Newly laid White Leghorn eggs were injected, cultured and hatched as previously described [26]. Genomic DNA from the heart and ovarian tissues of 22 two-month-old G_0_ hens were PCR screened, and the results showed that 9 (40.9%) G_0_ hens were integrated with hLY fragment in their heart tissues while 20 (90.9%) G_0_ hens were integrated with hLY fragment in their ovarian tissues. Semen DNA was extracted from adult males and screened. PCR and Q-PCR analyses were conducted to estimate the copy number of the hLY fragment in the germ line. The results of these analyses demonstrated that the frequencies of vector sequences in the examined semen ranged from 0.1% to 10% (Fig. 3, Table 1). hLY-positive males were mated with wild-type Leghorn hens to generate G_1_ birds.

**Table 1:** Q-PCR analysis of semen DNA from hatched male chickens and the efficiency of germline transmission.

Viral titre (TU/ml)	G_0_ Bird no.	Semen copy number	G_1_ trasgenic offspring/total	Sex of positive G_1_
**10** ^**9**^	E00127	0.025	2/88(2.27%)	1♀1♂
**10** ^**10**^	E00135	0.090	0/84	—
**10** ^**10**^	E00137	0.005	1/311(0.32%)	1♂
**10** ^**10**^	E00149	0.028	1/60(1.67%)	1♂
**10** ^**10**^	E00174	0.098	1/169(0.59%)	1♀
**10** ^**11**^	C00650	0.083	0/118	—
Total	—	—	5/830(0.60%)	2♀3♂

### The germline transmission of transgenes

**Fig 4:**
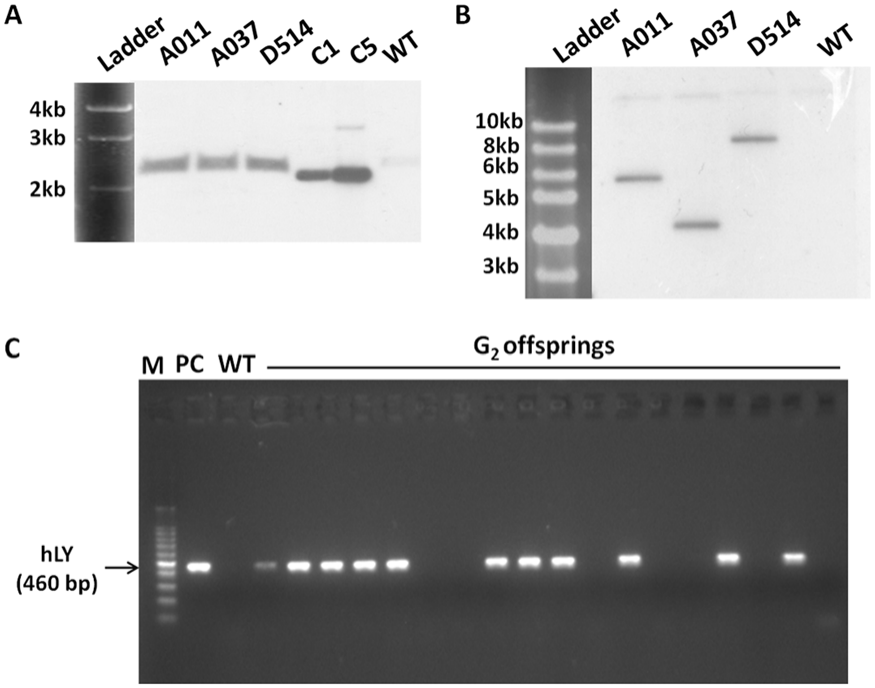
The identification of transgenic chicken. (A, B) Southern blot analyses of genomic DNA from individual G1 birds. Genomic DNA samples (10 μg) were digested with *Pvu*II(A) or *Nde*I (B) to generate either a fragment (2.3 kb) spanning the vector or a junction fragment between viral and chicken genomic sequences respectively, then hybridised with a probe for hLY. (C) PCR analysis of genomic DNA from blood of G_2_ offspring sired by A011. A011, A037 and D514, G_1_ offspring of G_0_ birds injected with pWPXL-EREOV-hLY. WT, wild-type White Leghorns. C1, plasmid control with a copy number of 1. C5, plasmid control with a copy number of 5. PC, positive control.

**Fig 5:**
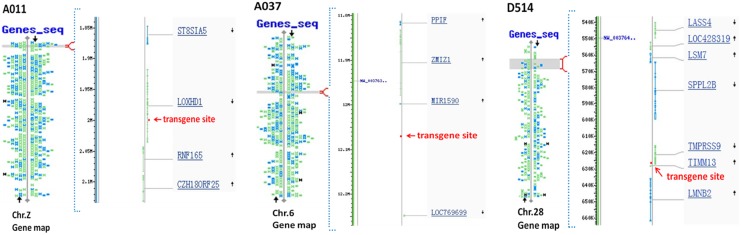
The identification of genomic integration sites in G_1_ transgenic birds by genome walking. The alignment of sequences produced by genome walking was analysed using the BLAST database of assembled genomes (http://www.ncbi.nlm.nih.gov/) and mapped on the chicken genome.

Six G_0_ semen-positive males were chosen for crosses with wild-type hens. A total of 830 chicks were screened by PCR, and five (0.60%) transgenic G_1_ offspring were identified. In Table 1, we listed the transgenic G_1_ offspring from individual G_0_ males and the corresponding copy number of hLY in the G_0_ semens analysed by Q-PCR. The efficiencies of germline transmission were lower than the frequencies at which vector sequences were present in examined G_0_ semen samples. Two of the five G_1_ transgenic chicks died less than one month after birth. The remaining three transgenic chicks included two males (A011 and D514) and one female (A037). The insertion of a single copy of the vector sequence in each of these chicks was confirmed by Southern blot analyses with *Pvu*II digested gDNA to generate a fragment (2.3 kb) spanning the vector (Fig. 4A) and *Nde*I to generate a junction fragment between viral and chicken genomic sequences (Fig. 4B). Genome walking analyses of these three birds revealed that the transgenes were randomly integrated into chicken chromosomes (Fig. 5, Table 2).

**Table 2:** Chromosomal integration sites of G_1_ transgenic birds.

Bird no.	Sex	Vector	Insertion site	Insertion region	Gene name
A011	♂	pWPXL-EREOV-hLY	Chr(Z):2007576–2007577	Intron	LOXHD1
A037	♀	pWPXL-EREOV-hLY	Chr(6):12078789–12078790	Intergenic	
D514	♂	pWPXL-EREOV-hLY	Chr(28):626355–626356	Exon	TMPRSS9

One of sexually matured G_1_ transgenic cocks, A011, was mated with wild-type Leghorn hens to generate G_2_ birds. The PCR screening of the genomic DNA of G_2_ offspring confirmed that 54 chicks including 24 males and 30 females among 111 total G_2_ birds were hLY-positive (Fig. 4C). The efficiency at which transgenes were transmitted from G_1_ males to G_2_ offspring was 48.6%.

### Transgene Expression Analyses

**Fig 6:**
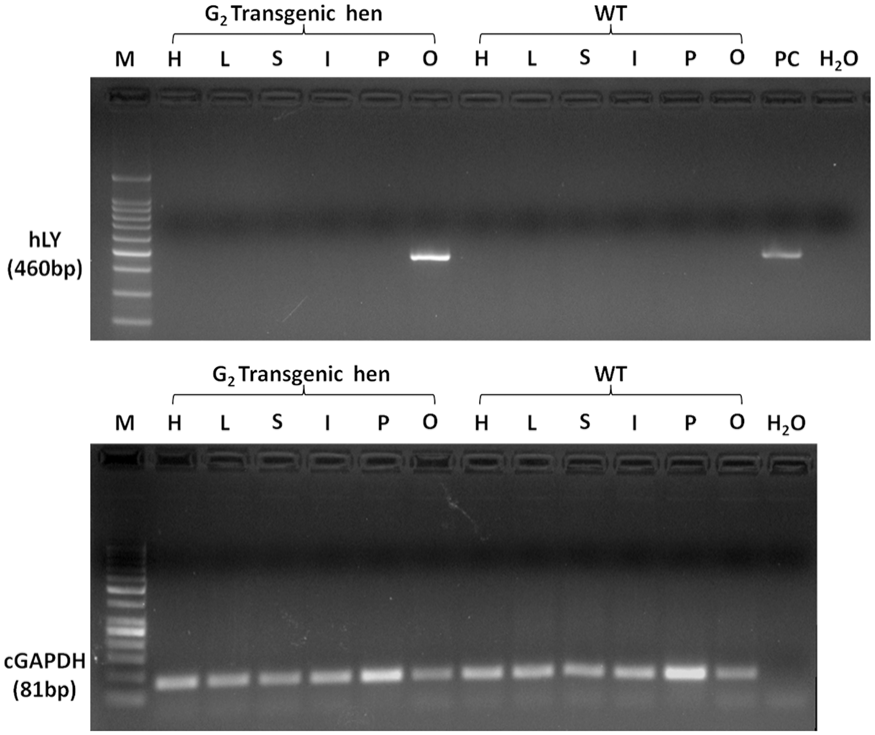
RT-PCR analysis of total RNA from G_2_ hens. Total RNA was extracted from the heart (H), liver (L), spleen (S), intestine (I), pectoralis (P) and oviduct (O) of the adult G_2_ transgenic hen and non-transgenic hen. RNA expression of hLY were detected from each sample. Chicken GAPDH was used as an internal control for RT-PCR. PC, positive control.

**Fig 7:**
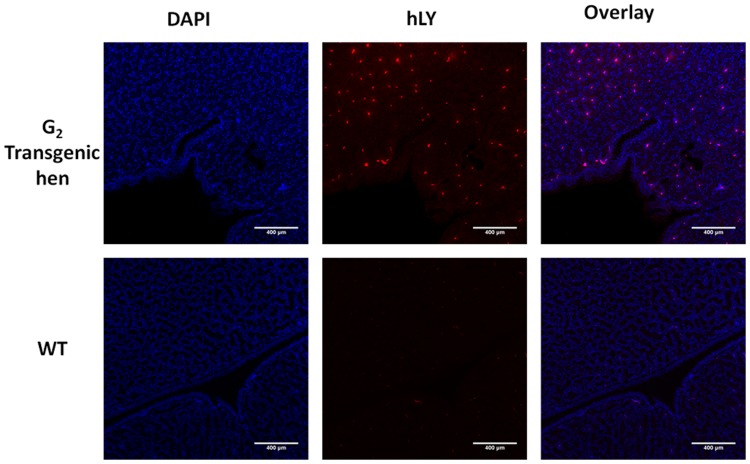
Immunofluorescence detection of hLY in oviduct sections. Sections of the magnum portion of the oviduct of G_2_ transgenic hen which was the offspring of G_1_ transgenic rooster A011 and a wild type White Leghorn were immunolabeled with an anti-hLY antibody. The nuclei are stained with DAPI and hLY staining is in red.

Tissue samples were collected from heart, liver, spleen, intestine, pectoralis and oviduct of the adult G_2_ transgenic hen and non-transgenic hen to assess RNA expression of hLY. The results showed that transgene expression of hLY was restricted to the oviduct of adult transgenic hen (Fig. 6). Immunofluorescence analysis of sections of the magnum of the oviduct of the adult G2 transgenic hen and non-transgenic hen revealed that rhLY protein was expressed in the oviduct of transgenic hen, coincident with the RT-PCR analysis results (Fig. 7).

**Fig 8:**
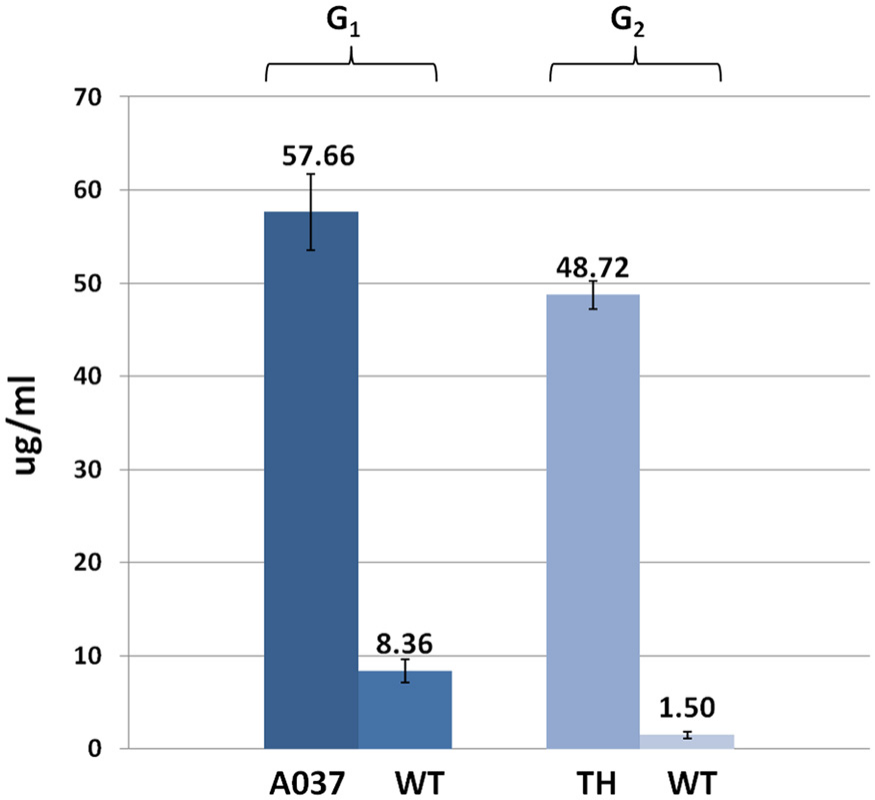
The concentration of recombinant human lysozyme in the egg whites from transgenic hens.

The whites of 10 consecutive eggs from the only G_1_ transgenic hen with this vector, A037, and the G_2_ transgenic hen sired by A011 were collected and mixed. ELISA was used to assess the presence of rhLY in the resulting mixture; as a control, egg whites from non-transgenic hens were also assayed. The mean concentration of rhLY in the whites of eggs from G_1_ transgenic hen A037 was 57.66 ± 4.10 μg/ml and from G_2_ transgenic hen sired by A011 was 48.72 ± 1.54 μg/ml. Low concentrations of hLY was detected in egg whites from non-transgenic hens; this phenomenon likely reflected low levels of cross-reactivity resulting from the similarities between hLY and cLY, which is naturally present in eggs (Fig. 8).

ELISA was utilised to assess the presence of recombinant human lysozyme in samples of egg whites from G_1_ and G_2_ transgenic hens and from non-transgenic hens. A037, G_1_ transgenic hen. TH, G_2_ transgenic hen sired by A011. WT, wild-type White Leghorns.

## Discussion

For several decades now, different strategies have been used in the production of transgenic chickens. Among the reported methods for genetic modification of avian germ cells, injection of lentiviruses and transfer of PGCs appear to be the most effective and commonly used. In comparison with the lentiviral methods, the PGCs approaches provide a better accessibility for the sophisticated modifications of the PGCs in culture and allow for in vitro screening of genetically modified PGCs. Recently, more subtle mutations and gene targeting have been achieved in chickens by modifying cultured PGCs with transposons or transcription activator-like effector nuclease (TALEN) [29, 30, 36, 37]. However, the processes of long-term propagation of PGCs, the generation of stable transfected PGCs, and the reinjection of PGCs into recipient embryos are highly skilled and costly, making it difficult to apply in general laboratory.

In this study, we constructed a plasmid based on a replication-defective HIV-1 construct containing the cDNA sequence of the human lysozyme gene. In this plasmid, the human lysozyme gene was driven by a 2.8-kb regulatory sequence from the chicken ovalbumin gene, as described by Lillico [34]. By microinjecting the viral vector into the sub-germinal cavity of stage X chick embryos, chimeric transgenic birds could be produced with germline transgene copy numbers of approximately 0.001–0.1. The incorporation of the human transgene into these birds was confirmed by PCR and Q-PCR analyses of semen DNA from G_0_ males.

The practical efficiencies at which transgenes were transmitted from G_0_ males to G_1_ offspring were lower than expected levels. For example, the copy number of hLY in the semen of one of the founder roosters, E00174, was 0.098; thus, among 100 G_1_ progeny of this rooster, 10 hLY-positive birds would be expected. However, in actuality, we found only one hLY-positive bird among the 169 G_1_ progeny of E00174. This deviation from expectations may have been caused by a number of factors. For instance, the motility of the transgenic sperm might be reduced by the random insertion of the lentivirus; alternative, the transgene might inhibit a gene associated with embryonic development, causing transgenic embryos to die during hatching (we did not examine dead embryos). With respect to the transmission of the transgene from G_1_ to G_2_ (data from A011), the ratio of transgenic offspring to total offspring was 54/111 (48.6%). This ratio did not significantly differ from the expected Mendelian ratio, indicating that the Mendelian inheritance of individual alleles occurred.

The human lysozyme structural gene, which contains four exons and three intervening sequences, has a total length of 5856 bp [38]. Given the capacity of lentiviral vectors (approximately 9 kb) and the fact that virus titres decline as donor sequence lengths increase [26], we used the cDNA sequence (447 bp) of the human lysozyme gene in this study. Titres of approximately 10^11^ TU/ml could be obtained for the vector pWPXL-EREOV-hLY, which has a total length of 6.5 kb. Three hLY-positive lines with different transgene integration sites were obtained. rhLY was expressed at 57.66 ± 4.10 μg/ml in the whites of eggs from A037, the only female G_1_ transgenic bird produced in this study. A similar hLY expression level (48.72 ± 1.54 μg/ml) was observed in the lines produced by A011, which featured transgene insertions in an intron of the LOXHD1 gene. hLY and cLY have highly similar primary and tertiary structures [39]; this similarity might explain why we detected low hLY concentrations in ELISA analyses of non-transgenic eggs.

A 2.8-kb chicken ovalbumin promoter was used to drive the transgene expression, which from the chicken ovalbumin gene regulating the ovalbumin protein expression to the oviduct of laying hens. Data from the RT-PCR and immunofluorescence analysis of G_2_ hens indicated that the expression of the rhLY in terms of tissue-restricted expression. The observation that the use of a lentiviral vector to deliver a transgene driven by a 2.8-kb chicken ovalbumin promoter produced transgene expression in the hens’ eggs is consistent with the findings of previous reports [34, 39, 40].

Human lysozyme is more active than chicken egg white lysozyme; in addition, studies have demonstrated that the addition of hLY to mouse milk (380 μg/ml) and dairy goat milk (270 μg/ml) by genetic engineering effectively inhibited several bacterial strains [15, 41]. Thus, the addition of hLY to eggs might produce similar bacteriostatic effects and therefore potentially prolong the shelf life of edible eggs.

There are significant advantages of using hens as bioreactors in comparison with the utilization of cattle, sheep or goats, including the short incubation and generation time, appropriate glycosylation of proteins and the nature sterile contents of eggs, which making the chicken oviduct bioreactor is more attractive for commercial production. We have shown that we can successfully obtain germline transgenic hens with oviduct-specific expression of recombinant human lysozyme by microinjection of lentiviral vectors and the recombinant human lysozyme proteins have been demonstrated to be able expressed stably in egg whites of transgenic hens after germline transmission. The study described here could be a contribution of the possibility of using transgenic birds for the production of therapeutic proteins or other valuable proteins.

## Supporting Information

S1 Data File contains Data A and Data B. (DOCX)

S1 Fig The RT-PCR and Western blot analysis of the modified hLY. (A) RT-PCR analysis of the modified hLY expression in transduced cells. (B) Western blot analysis of the modified hLY. The modified hLY sequence was cloned into the plasmid pBudCE4.1. This plasmid was transduced into four different cells: HEK293T, BHK, HT1080 and DF-1 (labeled as 293T-hLY, BHK-hLY, HT1080-hLY and DF1-hLY), allowing the biological function of the modified hLY to be estimated. The GFP sequence was cloned into the plasmid pBudCE4.1 and transduced into the four different cells (labeled as 293T-GFP, BHK-GFP, HT1080-GFP and DF1-GFP) as parallel controls. The untreated cells (labeled as 293T-NC, BHK-NC, HT1080-NC and DF1-NC) were negative controls. PC, positive control. Human GAPDH was used as an internal control for RT-PCR; β-actin was used as an internal control for western blotting.
(TIFF)

S1 Table Primer List. (DOCX)
