# Supplementary material for: Expression of Recombinant Human Lysozyme in Egg Whites of Transgenic Hens
Source: PLoS One. 2015 Feb 23;10(2):e0118626. doi: 10.1371/journal.pone.0118626 (PMC4338068; doi:10.1371/journal.pone.0118626)
Supplement: S1 Data — (DOCX) [file pone.0118626.s001.docx]

**Supporting Information**

**Supplement Data A: The sequence of cloned 673-bp ERE**

tctagagagagtaagcaacaatcttcttgcagttggaaggttacctgggataggtggcaatggattgccctgcccagcacagctgtgcaaagcagtacaaatagttttgtcacacattgtttgacaatgcttgtcccaagaaaaggtcagctaaggctctgctgccctttcctatgccaggcatttcattgtgggtctgtccctaaaccaacagtctcatgaataaagactaggagacctgaaagttataaaagcactttttatccaaaaggatatgaagtccaggtgagctcacaggtcaaagcctcttatccaatcactaaaacctactcagaacttgagattttgttctcctctttgccactacaattttgacattaggaaggagcacattaattgttctctgtttgtctttgttgcatattgtgagtgtggaaattgccttttcatgagggaaagaagcaaccattcatggagagagaaatcattttgctcaggttacagatgaatgtctaatggattgtcattgcataccttggtgctgtagccatcagtgatggtttctctctcagctgttttgtgggattgtgttgtttcccgtttgaagaaaatcaggtatcaggtcaagctcctttggatgtgagttcatagtccacctggcatttttctgcag

**Supplement Data B: The sequence of cloned 2.8-kb OV promoter**

ggtgaactctgagttgtctagagcaaacagcagaacagtgaaaatgtaaggatggaatgctgtacatagtaccatgcagggtactctatggtaggctacaacagtaaattacgagcagtttttaggcaattaaatgttaacaagtagttttaaagtaattctgtggtaatgtgtctgttgctatatccacctctcatgtgcatgttcaaaaccatattcataaatctatttatgtatttgcattcagttgtcttttgggtagcaaactgtcccagaagccagttgcctctacatatttttgttcagtgaaagctagaattcattgatacttttcagtacctctgattaaaacacaatctgataggcttgcaaaactggaaattcaaagagcaaatttcagtaaactttaggtttggacagatatatgagaaagcagaggcttgctgactattttatttcttatttttattccctaaaaataaatgtagagaaatatctgtttgttgcacactacttgctatgagtagatcttcaaaagtatttttacctttgttttggtgatggcagaatagataaggaatgtaatttatatggggtcatgtagtctaggagaaagacacgcatgtaattcatattctgctctattgcactttcaggtatggtttgctttgctcaaagatatgcatgtgtactgtagtataaactttctgtggagttaaattttagtggtgacattcagacagaagagaaatgcagacatgataaaatagcaatgtttactataaaacagagccactgaatgaattcttgttcatgacatagaccaatagaagatttatacttgttctgtctgtttctattataaagagctgaactgtacaactattgtatagccagtgtgcttatataaagcacagcttttggagccagcatgaatctagttgctttcctgagatttatataatctgtgaaagtcagaagtccttcagagcccagccctttatatgcgtactgagtgctggggcctcaggattggattttctgtattaaacccctcaaaagtttttactgaccacgtgtgtgagtatacacacacacatttttctcattttcttttctgtatataagttcacatgtatctattattgtaagaatatacgtttatgcaccccccacatttttatcttgtgtagtgatcagcagctgcactttgcaggaattaaacttctagagaattttcacattaaaataactccccagaattcactgaacaccatgattttgctctctgtgcactctgtagggctagaagttaatcaagcaaactgcaaagcatatcagatagtgaacgacaggataagatgttctgaaattaaaaacatattttaagcacaaagaataagcctcctgaaaacaaacacaaagcttttacacataataaaatagtgcagaatgcatacacaggtgagaagtttttatagggggtatcacgcaggtacttcacccttaaagatacaacacatagcacaataattgttaattttttaaagtttaggtgcaagtaagagctaatatagagagaaggtaattccagagagttgcttacctttcgagcttgactgctaaaggcaatacagctttctagctgtatgtacagacactggctgagccctggggaatatatagtctgaattgtgacccacccacaggttcccttcagaagtttgacctttgacaccatagaaatcatttaatgggattgggttagattttagtttcaataggtccattttggattgaatggagagcaaatattagtttttaattctgggtaacaatgtgttttctgcctgttctgctaatccatcaggactgttggatgggagagaagactgggaaatattgctcatgttccattgagcttcagttacaaccagataatgggatctttaagaaaacagaaaaatgtgggaaccttggagatggaaaacataattagcaattattagttagtgtgcttattactatggttgtagtaacagaccagaagtctgtttcatttgatccttcttgtatgtacaatgtgcatctgagccacgctagacaggacataaatgagaacaagacttgacctattattttcttgacaaaataggagaaataaagaagcgtgcatgtgaaggagccaactgagactagagtgaagagcagacacactttctttcctatagttggaatatttaaatctatctttttatgggtgtgaatgctttataacaaacttttattctgaggatacagcaaaacatagctccatacaatgcaaaacaatactcaatttcaaatgtgtttatgatatgaacttgcagtgttcctcaaagatcttccatgaataacttaatggcctggcagatgacagaggaattgtgaaattcagctggaggagtgttcatggttcgagggacaatcataatatacaatagcaaatatatttcagttatagaagctattgttctgtattgaaataatagaattgacaaacagtaaagaaaccattctgacctctgtaaagcactgtttgatttaaaaatgggggaaaaaagtacaacataattcttcaggacatacatagagatcactgcaatctctgttaagcagaattactttcctataccactagctgaagtttagtcagtgccattttcttttgtttctctccttccttttgtgaaaacatatatactgtggaaatctacattctccttgccaagtctgaggacttaag
