# Supplementary figures and images for: Expression of Recombinant Human Lysozyme in Egg Whites of Transgenic Hens
Source: PLoS One. 2015 Feb 23;10(2):e0118626. doi: 10.1371/journal.pone.0118626 (PMC4338068; doi:10.1371/journal.pone.0118626)

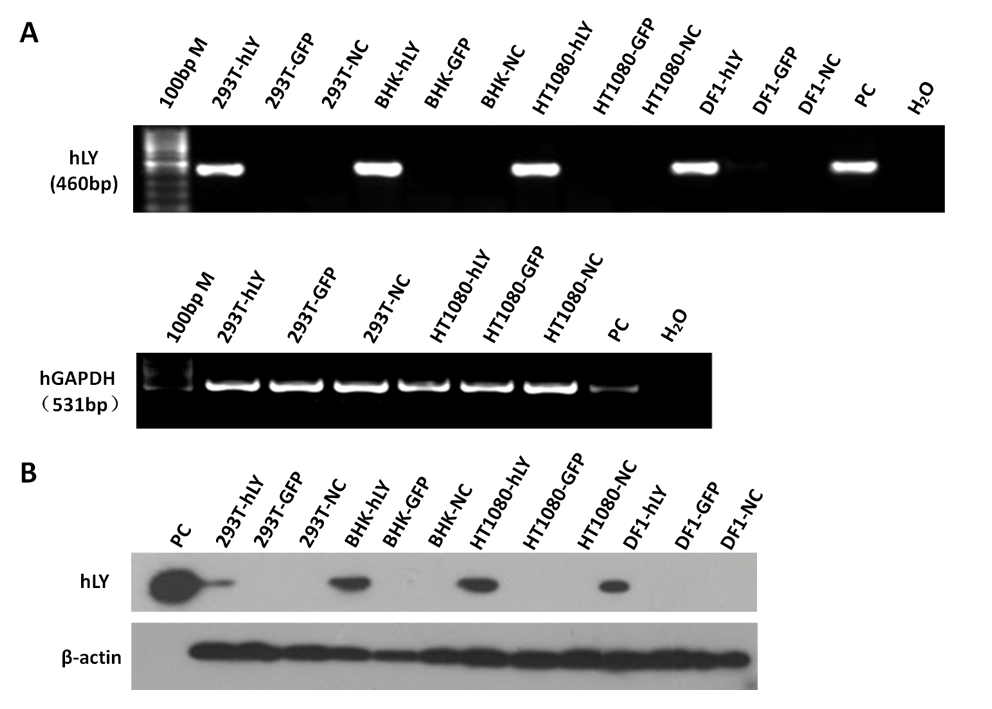

Supplement: S1 Fig — (A) RT-PCR analysis of the modified hLY expression in transduced cells. (B) Western blot analysis of the modified hLY. The modified hLY sequence was cloned into the plasmid pBudCE4.1. This plasmid was transduced into four different cells: HEK293T, BHK, HT1080 and DF-1 (labeled as 293T-hLY, BHK-hLY, HT1080-hLY and DF1-hLY), allowing the biological function of the modified hLY to be estimated. The GFP sequence was cloned into the plasmid pBudCE4.1 and transduced into the four different cells (labeled as 293T-GFP, BHK-GFP, HT1080-GFP and DF1-GFP) as parallel controls. The untreated cells (labeled as 293T-NC, BHK-NC, HT1080-NC and DF1-NC) were negative controls. PC, positive control. Human GAPDH was used as an internal control for RT-PCR; β-actin was used as an internal control for western blotting. (TIFF) [file pone.0118626.s002.tiff]
