# Supplementary material for: Expression of Recombinant Human Lysozyme in Egg Whites of Transgenic Hens
Source: PLoS One. 2015 Feb 23;10(2):e0118626. doi: 10.1371/journal.pone.0118626 (PMC4338068; doi:10.1371/journal.pone.0118626)
Supplement: S1 Table — (DOCX) [file pone.0118626.s003.docx]

**Table S1 Primer List.**

| Name | Sequences (5′-3′) | Type |
| --- | --- | --- |
| 2.8kOV-F | CTTAAGTCCTCAGACTTGGC | PCR |
| 2.8kOV-R | GCCCCGGGTGAACTCTGAGTTGTCTAG | PCR |
| ERE-F | CTGCAGAAAAATGCCAGGTGG | PCR |
| ERE-R | TCTAGAGAGAGTAAGCAACAATCTTCT | PCR |
| cGAPDH-F | TAGAGGCTGGCAGTTCTGGT | PCR |
| cGAPDH-R | CAGTCCCAGCCCATACAACT | PCR |
| Qsex-F | CTATGCCTACCACATTCCTATTTGC | PCR |
| Qsex-R | AGCTGGACTTCAGACCATCTTCT | PCR |
| hLY-F | TGTACGACACTGGCAACATG | PCR |
| hLY-R | CACTCCACATCCCTGAACATA | PCR |
| QPCRF2 | GGAGAGCGGATACAACACAAG | Q-PCR |
| QPCRR2 | CCAGTAGCGGCTATTGATCTG | Q-PCR |
| RT-cGAPDH-F | CGATCTGAACTACATGGTTTACATGTT | RT-PCR |
| RT-cGAPDH-R | CCCGTTCTCAGCCTTGACA | RT-PCR |
| HLY-F119 | GAATGAGCCTGGCAAAC | Southern Blot |
| HLY-R444 | CACTCCACATCCCTGAAC | Southern Blot |
| WPRE-SP1 | TGTTGGGCACTGACAATTCCG | Genome Walking |
| WPRE-SP2 | CTCAATCCAGCGGACCTTCCTT | Genome Walking |
| WPRE-SP3 | TCAGACGAGTCGGATCTCCCTT | Genome Walking |
